# Moxibustion Ameliorates Ovarian Reserve in Rats by Mediating Nrf2/HO-1/NLRP3 Anti-Inflammatory Pathway

**DOI:** 10.1155/2021/8817858

**Published:** 2021-05-27

**Authors:** Ge Lu, Qian Wang, Zi-Jing Xie, Shang-Jie Liang, Hong-Xiao Li, Ling Shi, Qian Li, Jie Shen, Jie Cheng, Mei-Hong Shen

**Affiliations:** ^1^College of Acupuncture Moxibustion and Tuina, Nanjing University of Chinese Medicine, Nanjing 210023, China; ^2^Key Laboratory of Acupuncture and Medicine Research of Ministry of Education, Nanjing University of Chinese Medicine, Nanjing 210023, China

## Abstract

Diminished ovarian reserve (DOR) is an increasingly emerging reproductive disorder that disturbs reproductive-aged women, which is closely linked with inflammation. In clinic, moxibustion has already been applied for reproductive problems. In the present study, we examined the involvement of inflammation in DOR and investigated the effect of moxibustion for its anti-inflammatory activities. *Methods*. DOR rat model was established using tripterygium glycosides A tablets (TGs) suspension by intragastric administration and was then treated with either moxibustion or hormone replacement therapy (HRT), respectively. Estrus cycles were observed through vaginal cytology. Ovarian morphological alterations were observed by HE staining. The serum levels of follicle-stimulating hormone (FSH), estradiol (*E*_2_), anti-Müllerian hormone (AMH), tumor necrosis factor alpha (TNF-*α*), and interleukin-10 (IL-10) were measured through ELISA. The expression levels of Nrf2, HO-1, and NLRP3 were detected using immunohistochemistry. Nrf2, HO-1, and NLRP3 mRNA were examined by RT-PCR. *Results*. Moxibustion improved estrus cycles, FSH, *E*_2_, and AMH levels relative to DOR rats as well as HRT, while also inhibiting ovarian tissue injury. Anti-inflammatory cytokine IL-10 in peripheral blood was upregulated, and proinflammatory factor TNF-*α* was decreased after treatment with moxibustion. Moxibustion enhanced the expression of mRNA and protein of nuclear factor erythroid 2-related factor (Nrf2) and heme oxygenase-1 (HO-1); in the mean time, nucleotide-binding oligomerization domain-like receptor protein 3 (NLRP3) was suppressed. *Conclusions*. We demonstrated that moxibustion could ameliorate the ovarian reserve in rats induced by TGs. Overall, the effect of moxibustion was comparable to that of HRT. The underlying mechanism could be attributed to the anti-inflammatory effects of moxibustion, which suppressed NLRP3 activation by upregulating Nrf2/HO-1 signaling pathway.

## 1. Introduction

Diminished ovarian reserve (DOR) is one of the main causes of female infertility, which leads to premature reproductive senescence. DOR is characterized by increased follicle-stimulating hormone (FSH) level, low anti-Müllerian hormone (AMH), and low antral follicle counts at women's reproductive age [1]. For women who suffer infertility, the prevalence of DOR reaches to approximately 10% [2]. Beyond procreative concerns, DOR has adverse implications in multiple body system, including bone functions, cardiovascular, and nervous system [3].

Inflammatory signaling was closely linked with ovarian follicle depletion [4, 5]. Inflammation is a nonspecific immune response of vascular tissue to infection, injury, or other harmful stimulation. In fact, inflammatory process is necessary for appropriate follicular development and many physiologic reproductive processes. Particularly, ovulation displays hallmark signs of inflammation, but that excessive inflammation may contribute to poor oocyte quality or abnormal ovulation [6, 7].

Nucleotide-binding oligomerization domain-like receptor protein 3 (NLRP3) inflammasome is one of the nucleotide oligomerization domain (NOD)-like receptor proteins that respond to pathogen-associated and damage-associated environmental irritants. Local or systematic inflammatory reactions can activate the NLRP3 inflammasome, which is widely distributed in different tissues and play a fundamental role in innate immunity. Inappropriate activation of NLRP3 inflammasome subsequently induces more proinflammatory cytokine release, such as tumor necrosis factor alpha (TNF-*α*) and interleukins, which, in turn, further aggravates inflammatory response and the progression of inflammatory disorders [8–11]. However, clinical drugs targeting the NLRP3 inflammasome are still unavailable [12]. Nuclear factor erythroid 2-related factor 2 (Nrf2)/heme oxygenase-1 (HO-1) pathway is related to the activation of NLRP3 inflammasome in several inflammatory diseases [11, 13]. NLRP3 was highly expressed when Nrf2 was silenced [11].

Moxibustion, which has a positive effect on ovarian function, is increasingly accepted as a complementary and alternative medical therapy in traditional Chinese medicine that has a positive effect on ovarian function [14–17]. Lines of evidence showed that moxibustion could reduce the inflammatory cascade reaction and inflammatory injury by controlling inflammasome activation [18]. Here, we speculated that moxibustion could depress DOR in rats through restricting NLRP3 transcription by activating Nrf2/HO-1 signaling pathway.

## 2. Materials and Methods

### 2.1. Animals

Eight-week-old female Sprague-Dawley rats (190 ± 10 g) were purchased from Shanghai Xipuer-Bikai Lab Animal Co., Ltd (license number: SCXK (Hu) 2018–0006). All rats were housed in standard polypropylene cages for five rats per cage and kept under 24–26°C and under humidity of 50–60% with 12 : 12 hours light-dark cycles. Water and food were free. After 10 days of observation, only 40 rats with normal estrus cycles were accepted; those with irregular cycles were used for other experiments. The investigation followed the National Institutes of Health Guidelines for the Care and Use of Laboratory Animals (NIH publications, version 1996) and the Animal Research: Reporting In Vivo Experiments (ARRIVE) Guidelines. All rats' experimental protocols and procedures were approved by the Animal Ethics Committee of Nanjing University of Chinese Medicine Laboratory Animal Center under a project license (no.: 201909A027).

### 2.2. Experimental Design

**Figure 1:**
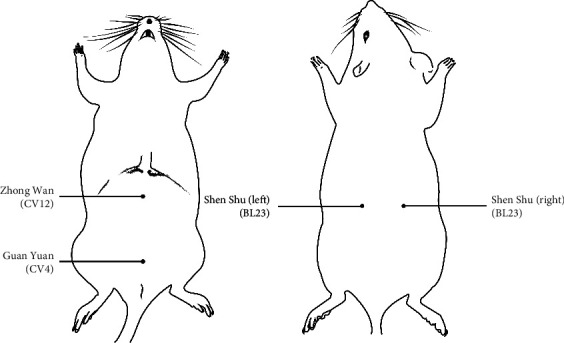
The acupoints of moxibustion. Two sets of acupoints were used alternatively; “Zhong Wan (CV12)” and “Guan Yuan (CV4)” were located on the rats' stomach, and bilateral “Shen Shu (BL23)” was on their back.

**Figure 2:**
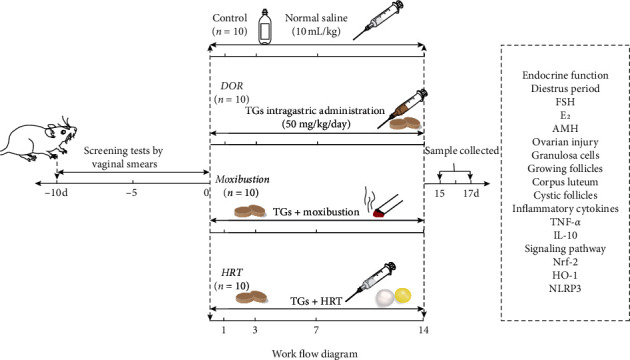
Work flow diagram.

Forty rats were divided into four groups by randomized digital table: control group (*n* = 10), DOR model group (DOR, *n* = 10), moxibustion group (*n* = 10), and hormone replacement therapy group (HRT, *n* = 10). The DOR rat model was established through intragastric administration of tripterygium glycosides (TGs) tablets suspension (50 mg/kg/day, SFDA approval no. Z43020138, Hunan Qianjin Co., Ltd., China) for 14 consecutive days as reported by previous studies [19, 20]. Rats in the control group were administered a physiological saline (10 mL/kg/day) for gavage. One hour after intragastric administration of TGs, those in the moxibustion group had a mild moxibustion with moxa sticks (diameter: 5.3 mm; length: 85 mm; Hunan Gosen Biological Co., Ltd., China) above bilateral, “Zhong Wan (CV12)” and “Guan Yuan (CV4)” (Figure 1 and Table 1) within 1 cm for 10 minutes each acupoint. These four acupoints were divided into two sets. One is bilateral “Shen Shu (BL23),” and the other is “Zhong Wan (CV12)” and “Guan Yuan (CV4).” The two sets of acupoints were used in turn with one moxibustion each day. Rats in HRT group were administered estrogen via the gastric route (0.1 mg/kg/day, SFDA approval no. J20130009, Bayer, Germany) from day one to day four, progestogen was added (0.8 mg/kg/day, SFDA approval no. H20031099, Bayer, Germany) on the fourth day, and no hormone replacement treatment was administered on the fifth day. This therapeutic cycle was repeated three times. Treatment of moxibustion and HRT followed intragastric administration of TGs one hour later. On the 15^th^ day, rats were anesthetized with 2% pentobarbital sodium (2.5 mL/kg, CAS: 57-33-0, Merck KGaA, Germany), which was intraperitoneally injected. 2 mL blood sample was taken from the abdominal aorta of each rat of group and then centrifuged in Gel tubes to collect serum for sexual hormone and inflammatory cytokines assay. Bilateral ovaries were excised. Afterwards, the right ovaries in every group were fixed in 4% neutral buffered paraformaldehyde to process paraffin blocks and then sectioned at 6 *μ*m thickness for histopathological studies. The left ones were used for testing mRNA expression. More intuitive experimental design is shown as work flow diagram (Figure 2).

### 2.3. Vaginal Cytology

Vaginal smears were taken at 8 : 30 am daily. Cast-off cells were collected to estimate the estrus cycles of rats. The average normal estrus cycle in a rat is 4–5 days, which can be generally divided into four stages: proestrus, estrus, metestrus, and diestrus. Different cell types appear and recede in each stage. Proestrus is characterized by a large number of nucleated epithelial cells and few leukocytes and lasts for 12–15 hours. Estrus duration ranges between 25 and 27 hours, which presents a large amount of cornified and irregular shaped epithelial cells without nucleus. Metestrus is characterized by a combination of nucleated epithelial cells, anucleated keratinized epithelial cells, and leukocytes and lasts for 6–8 hours. Diestrus is characterized by a large number of leukocytes and a low number of cornified epithelial cells and lasts for about 45–47 hours [21].

### 2.4. Enzyme Linked Immunosorbent Assay (ELISA)

Sexual hormone was measured using rat estradiol (*E*_2_), follicle-stimulating hormone (FSH), and rat anti-Müllerian hormone (AMH) ELISA kit (cat. no. RA20666, RA20044, RA20211, Bio-Swamp Life Science Lab, China) separately. Inflammatory cytokines were measured by rat tumor necrosis factor alpha (TNF-*α*) and interleukin-10 (IL-10) ELISA kit (cat. no. RA20035, RA20090, Bio-Swamp Life Science Lab, China). All experiments followed the protocols. Optical density (OD) was measured at 450 nm by enzyme labeling (ELX800, Bio Tek Co., Ltd, US).

### 2.5. Hematoxylin and Eosin (HE) Staining

Ten slides in each group were stained with HE. Morphology of ovaries, follicles, and corpora luteum was observed with light microscope (U-ND6-2 Fluorescence Multifunctional Microscopy, OLYMPUS, Japan). According to a previous study [22], follicles were classified as primordial follicle (a primary oocyte surrounded by a single layer of squamous follicular cells), primary follicle (a primary oocyte surrounded by a single layer or multilayered granulosa cells), secondary follicle (multilayered mass of granulosa cells with a fluid-filled antrum surrounding a larger primary oocyte), and Graafian follicle (GF), a secondary oocyte surrounded by the cumulus oophorus with large antrum.

### 2.6. Immunohistochemistry (IHC)

Three ovarian tissue sections in each group were incubated with the primary rabbit antibodies: anti-Nrf2 (1 : 50 dilution, cat. no. ab137550, Abcam Trading Co., Ltd., UK), anti-HO-1 (1 : 200 dilution, cat. no. ab13243, Abcam Trading Co., Ltd., UK), and anti-NLRP3 (1 : 50 dilution, cat. no.19771-1-AP, Proteintech Group Inc., USA) for 16 h at 4°C in a humidity chamber. Biotinylated secondary antibody anti-rabbit IgG (cat. no. SA1022, BOSTER Biological Technology Co., Ltd., China) was applied to the sections for 30 minutes and then incubated with streptavidin-biotin complex (SABC) for 30 minutes at 37°C. After DAB color development, images were captured under fluorescence microscope (BX60, Olympus Corp., Japan) at 400 magnification, and then, positive areas were analyzed using Image J software (Version 1.8.0, National Institutes of Health, USA).

### 2.7. Real-Time Polymerase Chain Reaction (RT-PCR)

Rat Nrf2, HO-1, and NLRP3 (ID number: Rn00582415_m1, Rn00561387_m1, Rn04244620_m1) were performed by TaqMan PCR technology (Applied Biosystems Inc., USA) to verify differential mRNA expression. Total RNA in 10 left ovaries of four groups were extracted using Trizol (cat. no.15596–026, Invitrogen Corp., USA) to synthesize template DNA by PrimeScript RT Reagent Kit (cat. no. RR037A, TaKaRa Bio Inc., Japan). The analysis was done in the 7500 Real-Time PCR system (Applied Biosystems Inc., USA). Results were analyzed with the comparative cycle threshold (CT) method and calculated from the △△CT values with the formula 2^−△△CT^. The relative quantitation value of Nrf2, HO-1, and NLRP3 was normalized to the level of GAPDH reference gene (ID number: Rn01775763_g1).

### 2.8. Statistical Analysis

Statistical analysis was performed by IBM SPSS statistics (version 22.0, SPSS Inc., USA). The rate of irregular cycles, number of days in each estrus phase, and area of positive cells were analyzed by chi-square test, and the results were reported as ratio and frequency. Quantitative data with a normal distribution and homogeneity of variance, including the concentration of sex hormone and inflammatory cytokines and relative expression of mRNA, was analyzed by one-way analysis of variance (ANOVA) among multiple groups, followed by the least significant difference (LSD) for the comparison of every two groups. The Kruskal–Wallis test was used for nonnormal distribution data and Tamhane's T2, variance nonhomogeneity. The results of quantitative data were expressed as mean ± standard deviation (*s*). *P* value of <0.05 was considered as statistically significant. *n* indicates the number of samples per group.

## 3. Results

### 3.1. Effect of Moxibustion on Endocrine Function

**Figure 3:**
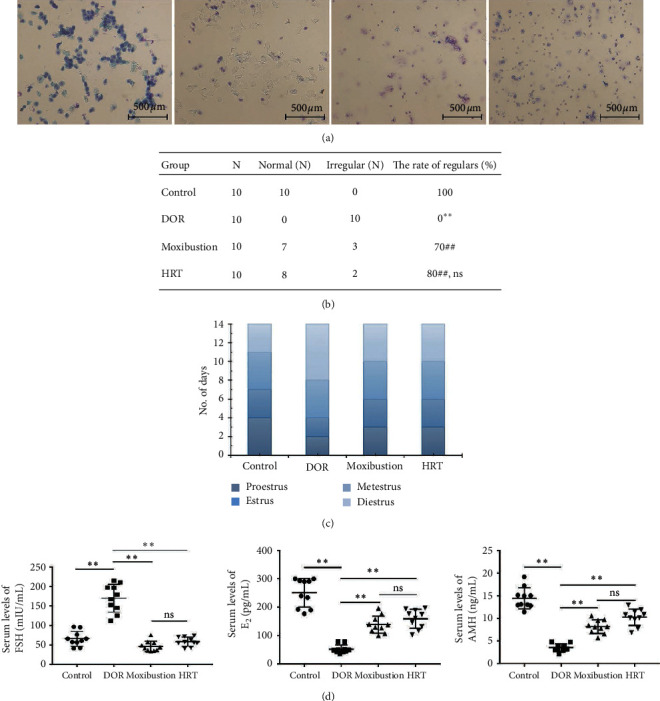
Effects of moxibustion on ovarian endocrine function. (a) Representative photographs of vaginal smears; from left to right: proestrus, estrus, metestrus, and diestrus (×200, scale bars: 500 *μ*m). (b) The rate of disturbance of estrus cycles (*n* = 10 each group). Compared with control group, *P* < 0.01; compared with DOR group, ^##^*P* < 0.01; compared with moxibustion group, *P* > 0.05; ns: not significant. (c) Representative number of days in each estrus stage. (d, e, f) The changes of sex hormone FSH, *E*_2_, and AMH in each group (*n* = 10 each group). Data was represented as mean ± s. *P* < 0.01; ns: not significant; *P* > 0.05.

Vaginal cytology was used to observe the changes of estrus cycles (Figure 3(a)). During the 14 days, diestrus prolonged and estrus phase decreased in DOR rats, but moxibustion and HRT reversed this trend where diestrus stages reduced and the days of estrus increased (Figure 3(c)). When treated with moxibustion or HRT, 70% and 80% of rats separately showed regular cycles (Figure 3(b)). The percentage of regular estrus cycles in moxibustion group was significantly higher than that in model group (*P* < 0.01). The results indicated that TGs had a negative effect on ovarian function, and moxibustion prevented the damage from TGs as well as HRT.

AMH, *E*_2_, and FSH serum levels were investigated by ELISA. Compared with that in the control group, the levels of AMH and *E*_2_ both declined (*P* < 0.01), while FSH concentration was increased (*P* < 0.01) in the DOR rats after 14 days of TGs lavage (Figures 3(d)–3(f)). However, moxibustion and HRT partly reversed the impact of TGs on sexual hormone endocrine as rats in these two groups showed a higher level of AMH and *E*_2_ (*P* < 0.01) and a decreased level of FSH than that in model group (*P* < 0.01). Moxibustion played a similar role to that of HRT on sex hormone (*P* > 0.05).

### 3.2. Moxibustion Prevented Ovarian Injury Induced by TGs

**Figure 4:**
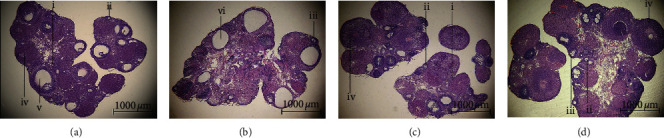
Effects of moxibustion on ovarian morphology (*n* = 10 each group). (a, b, c, d) Representatives of the four groups after hematoxylin and eosin (HE) staining (×40, scale bars: 1000 *μ*m). iv: corpus luteum after ovulation; i: primordial follicle; ii: primary follicle; iii: secondary follicle; v: Graafian follicle; vi: cystic follicle.

Ovarian follicles were contained in the cortex. Healthy follicles in different stages were observed in the control group (Figure 4(a)). In comparison with control group, ovaries in DOR rats showed more cystic follicles with thin-layered granulosa cells instead of Graafian follicles (Figure 4(b)). Nevertheless, moxibustion and HRT improved the situation characterized by more normal follicles with a multilayered mass of granulosa cells and fewer malformed follicles (Figures 4(c) and 4(d)). In moxibustion group, much more corpus luteum was filled with connective tissues and blood vessels, in which mature follicles had just been released (Figure 4(c)).

### 3.3. Effect of Moxibustion on Inflammatory Cytokines

**Figure 5:**
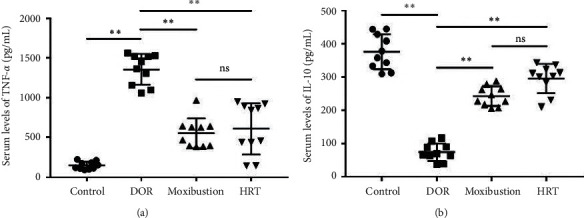
The changes of systemic inflammatory-related cytokines TNF-*α*; IL-10 (*n* = 10 each group). (a) The level of proinflammatory factor TNF-*α* in four groups. (b) The level of anti-inflammatory cytokine IL-10 in each group. All results of statistical analysis were expressed as mean ± s. *P* < 0.01; ns: not significant; *P* > 0.05.

To further investigate the inflammatory response in DOR rats, serum proinflammatory and anti-inflammatory cytokines were detected by ELISA. TNF-*α* is a proinflammatory cytokine, which regulates the immune response. We found that TNF-*α* was notably increased in the DOR group compared with the control rats (*P* < 0.01), indicating an upgradation of inflammatory level caused by TGs. On the contrary, the concentration of anti-inflammatory factor IL-10 was decreased in rats (*P* < 0.01). Then, we observed the influences of moxibustion or HRT on these inflammatory-related factors. It has been clarified that both moxibustion and HRT promoted the concentration of IL-10 (TNF-*α* level was significantly different after treatment (*P* < 0.01) (Figure 5).

### 3.4. Moxibustion Promoted Nrf2 and HO-1 at Both Transcriptional and Translational Levels and Inhibited Activation of NLRP3

**Figure 6:**
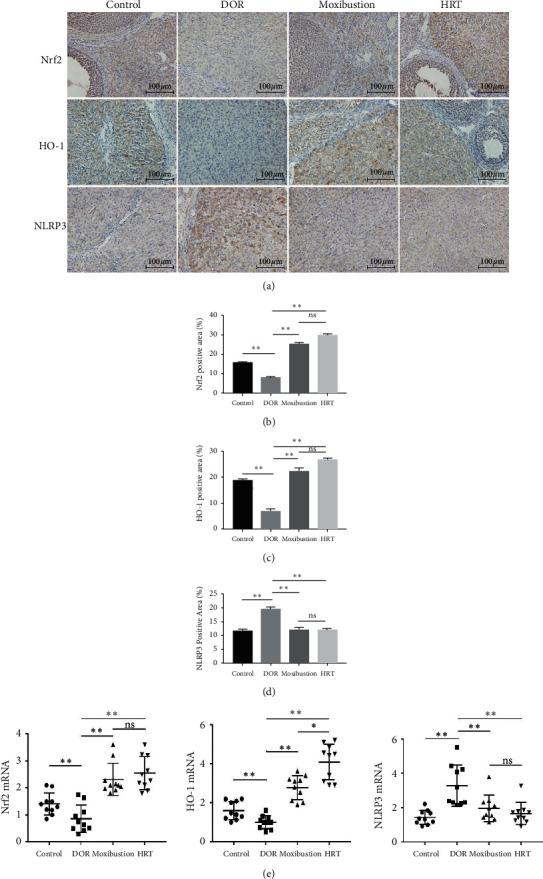
Effects of moxibustion on Nrf2, HO-1, and NLRP3 expression at transcriptional and translational level. (a) Representative images of ovarian with IHC staining of Nrf2, HO-1, and NLRP3 (*n* = 3 each group, ×400, scale bars: 100 *μ*m). (b, c, d) Quantitative analysis of Nrf2, HO-1, and NLRP3 positive area detected by IHC. The positive area was expressed as mean ± s. *P* < 0.01; ns: not significant; *P* > 0.05. (e) The relative expressions of Nrf2, HO-1, and NLRP3 mRNA were expressed as mean ± s (*n* = 3 each group). *P* > 0.05; *P* < 0.01; ns: not significant; *P* > 0.05.

We measured the mRNA expression of Nrf2, HO-1, and NLRP3 by TaqMan PCR, and their protein expression was evaluated through immunohistochemical analysis. At translational level, Nrf2 and HO-1 were apparently suppressed (all *P* < 0.01), while the level of NLRP3 was increased (*P* < 0.01) in DOR rats. NLRP3 level was significantly downregulated in the moxibustion group (*P* < 0.01), where, in the meantime, Nrf2 and HO-1 showed higher levels at the meantime (all *P* < 0.01). The previously mentioned findings were exactly opposite to those in model group (Figures 6(b)–6(e)). Moxibustion had a similar effect on Nrf2 and NLRP3 compared with HRT (*P* > 0.05). Under optical microscope, Nrf2 and HO-1 localized to cytoplasm of luteal cells and nucleus of granulosa cells was observed, and NLRP3 only presented in cytoplasm of granulosa cells (Figure 6(a)). Ovaries in control group shown larger positive area of Nrf2 and HO-1 than DOR rats, but more NLRP3 positive cells were found in ovaries of DOR rats (Figure 6(a)). There was no significantly difference between moxibustion and HRT group (*P* > 0.05).

## 4. Discussion

DOR means qualitative and/or quantitative decline of oocytes. DOR is usually accompanied by reproductive aging, which is a normal physiological phenomenon after a woman's mid-40s, but becomes pathological when it occurs at early 40s or even 30s [23]. DOR not only has a negative effect on the female reproductive lifespan but may also result in higher mortality if it develops into premature ovarian failure [24]. HRT is commonly used in clinic, but a meta-analysis suggested that HRT might increase the risk of ovarian cancer [25]. Under this premise, an effective treatment towards DOR with less side effects is urgently required.

Mild moxibustion is a noninvasive external treatment, producing comprehensive effects by physical and chemical influence [26]. The heat produced by burning Asiatic wormwood is a kind of infrared ray, which can get deep in body through the meridian system, stimulating the immune response [27]. Clinically, it is extensively used for reproductive disorders, owing to the warm and near-infrared stimulation, which was beneficial to the sex hormone level, ovulation rate, and pregnancy outcome for infertile women [17, 28]. Alternatively, moxibustion could attenuate inflammatory impairment via decreasing inflammatory cell infiltration, the anti-inflammatory effects of which have been proved in certain chronic inflammatory response [18, 29, 30].

The improving effects of moxibustion on ovarian reserve have been reported both in experiments and in clinical practice [15, 16]. *E*_2_ is secreted by growing follicles; thereby, the reduction of developed follicles may be the reason of lower *E*_2_ level in the DOR model group. The dropped level of *E*_2_ produced a negative feedback effect on FSH at pituitary level [31]. Besides, TGs directly reduces *E*_2_ synthesis in granulosa cells [32]. Therefore, the results, where DOR rats showed a high serum FSH level accompanied by a low *E*_2_ concentration, point to the dysregulation of ovarian secretion and a decrease of follicular reserve. Estrus cycle is another feature of ovarian function. The retention of estrus cycle in diestrus or metestrus indicates insufficiency of *E*_2_. The disorder of estrus cycles is considered as one of the earliest symptoms in the ovarian dysfunction [33], which is supported by results of estrus cycles and serum hormone in DOR rats. In the current study, moxibustion decreased the level of FSH and had a positive effect on *E*_2_ concentration. Actually, moxibustion increased AMH level, which performs as a direct marker for the size of growing follicle pool and is relatively independent of gonadal hormone circulation. As is shown in our study, moxibustion showed an almost similar effect to that of HRT in the protection of ovarian reserve and sex hormone secretion induced by TGs.

The activation of Nrf2-mediated pathway is one of the main defensive mechanisms of cells. Our previous studies have demonstrated that moxibustion could improve ovarian reserve in rats by Nrf2/HO-1 antioxidant pathway [34]. Recently, Nrf2 is gradually recognized as a central regulator of signaling pathways in inflammation [11, 35]. Inflammation participates in the decline of follicle quality and loss of quantity [6, 36], leading to the reduction of ovarian reserve. As an important component of immune system, NLRP3 inflammasome is one of the best-studied inflammasomes. It is an intracellular protein complex that initiates cellular injury after its assembly. Hence, inhibiting assembly of NLRP3 inflammasome is a target to alleviate inflammatory injury in associated diseases. Nrf2-mediated pathway as an upstream signaling has been proposed to play a pivotal part in the activation of NLRP3 inflammasome. Downregulation of Nrf2 increased the expression of NLRP3, so that NLRP3 inflammasome activation could be enhanced after Nrf2 silencing [11]. NLRP3 inflammasome contains a sensor protein nucleotide-binding domain- (NOD-) like receptor protein3 (NLRP3), an apoptosis-associated speck-like protein (ASC) and a zymogen procaspase-1. Under conditions of inflammation, tissue damage, cell death, reactive oxygen species, and metabolic stress, the assembly of a complete NLPR3 inflammasome catalyzes the conversion of procaspase-1 to active caspase-1 and releases downstream cytokines subsequently to aggravate inflammatory response [37]. It was found that TGs treatment caused cell apoptosis and inflammatory cell infiltration [38], and this local ovarian damage further led to the assembly of NLRP3 inflammasome. Data showed that moxibustion upregulated Nrf2/HO-1 expression and suppressed the level of NLRP3 at the same time, and the negative correlation between them in moxibustion group was the same as that in other groups, which is consistent with previous studies [11].

Furthermore, TNF-*α* and IL-10 were used to evaluate the degree of inflammation. The imbalance between proinflammatory and anti-inflammatory cytokines is involved in the apoptosis of granulosa cells and follicular atresia [39]. IL-10 is a key anti-inflammatory factor that plays a vital role in inhibiting the synthesis and release of certain proinflammatory cytokines. IL-10 application could not only alleviate local inflammatory response but also decrease the release of proinflammatory mediators, including TNF-*α* [40]. Upregulated TNF-*α* increases atresia follicles in ovaries [41], implicating in the development of infertility [42]. Similar to a previously reported study, the administration of TGs resulted in an increased inflammatory response, showing an elevation in serum TNF-*α* and a reduction in IL-10 [43], which was also demonstrated in our DOR rats. However, moxibustion regulates inflammatory response in two ways, including inhibiting the expression of proinflammatory cytokines and promoting anti-inflammatory factors [44].

## 5. Conclusion

Taken together, our findings here demonstrated that TGs damaged the ovarian tissue and the development of follicles, leading to the inappropriate activation of NLRP3 inflammasome. Furthermore, moxibustion activated Nrf2/HO-1 pathway, and then, it might downregulate NLRP3 to ameliorate the inflammatory microenvironment. The detailed mechanisms of the inhibition effect of moxibustion on NLRP3 inflammasome assembly by Nrf2/HO-1 pathway, particularly whether NLRP3 excessive activation is Nrf2/HO-1 dose-dependent, should be further investigated.

## Figures and Tables

**Table 1:** Acupoints and location.

Acupoints	Location
Shen Shu (BL23)	7 mm outward the 2nd lumbar vertebra, which is 4 vertebrae upward the 6th lumbar spine
Zhong Wan (CV12)	4/13 and 9/13 of the thoracic and pubic symphysis line on the midline of the abdomen, about 20 mm above the navel
Guan Yuan (CV4)	On the midline of the abdomen, when the distance between the midpoint of the 4th and 5th pairs of nipples ≥3 cm, Guan Yuan (CV4) is about 25 mm below the navel; when the distance between the midpoint of the 4th and 5th pairs of nipples <3 cm, Guan Yuan (CV4) is about 20 mm below the navel

## Data Availability

The data used to support the findings of this study have not been made available due to the nature of this research.
